# Diagonal integration of multimodal single-cell data: potential pitfalls and paths forward

**DOI:** 10.1038/s41467-022-31104-x

**Published:** 2022-06-18

**Authors:** Yang Xu, Rachel Patton McCord

**Affiliations:** 1 UT-ORNL Graduate School of Genome Science and Technology, University of Tennessee, Knoxville, TN 37996 USA; 2 Department of Biochemistry & Cellular and Molecular Biology, University of Tennessee, 309 Ken and Blaire Mossman Bldg 1311 Cumberland Ave, Knoxville, TN 37996 USA

**Keywords:** Data integration, Systems biology

## Abstract

Diagonal integration of multimodal single-cell data emerges as a trending topic. However, empowering diagonal methods for novel biological discoveries requires bridging huge gaps. Here, we comment on potential risks and future directions of diagonal integration for multimodal single-cell data.

With the advance of new single-cell technologies, single-cell computational analysis has moved into the multi-omics era. Integrating multi-omics single-cell data, therefore, has gained increasing attention from the single-cell community. This key research domain promises to help us understand complex cellular systems from different viewpoints, such as gene expression, chromosome structure, and even cellular imaging. Computational integration methods that match one modality with another can reveal a detailed picture of regulatory networks and cellular function (see Box 1 for important term definitions). However, different types of ‘omics data usually do not share the same features. For instance, transcriptomics describes expression of genes, while epigenomics measures histone modifications or accessibility across all regions of the genome. This feature discrepancy presents the first challenge to the development of integration methods. The other challenge stems from how single-cell data have been collected over the years. Though recent technologies enable multiple measurements to be made simultaneously on the same single cells (“joint-profiling”)^1–3^, most single-cell datasets profile different aspects of biology one at a time in independent groups of cells. Therefore, we lack ground truth about what is happening at the level of epigenetics, transcriptomics, and proteomics in the same single cell. This makes it difficult to evaluate the quality of proposed integration methods. In recent years, many integration methods have been published to address different scenarios of multimodal single-cell data integration. A recent key review summarized three major approaches of multimodal single-cell data integration and outlined published methods in each category^4^. Of these categories, “horizontal integration” methods require anchored features to align different modalities, while “vertical integration” methods need shared cells from multiple modalities as anchors. The “diagonal integration” approach requires neither anchoring cells nor features for integration, presenting a distinct advantage over horizontal and vertical methods. Because no prior knowledge is required, accurate diagonal integration is also challenging to achieve. Despite the rapid increase in new diagonal integration methods, there is not a single diagonal method that has been extensively examined and carefully benchmarked for its utility in multimodal integration in complex cellular systems.

## Box 1

Schematic of horizontal, vertical, and diagonal integration and key term definitions.

Examples of different modalities (RNA-seq and ATAC-seq) are indicated by color while feature type (gene or region) is indicated by shape. Dotted lines indicate how features or cells are matched in different types of integration.



## Goals and common features of diagonal integration approaches

In this comment, we focus solely on diagonal integration. Over the past 3 years, there has been a steady increase in publications describing new diagonal methods for the integration of multimodal single-cell data^5–11^ (Supplementary Table 1), indicating strong interest in the unique advantages of diagonal integration. Since horizontal and vertical methods require either anchored features or anchored cells, their application is limited to cases where it is feasible to engineer matched features (which is often quite difficult, particularly with disparate measures such as cell imaging and gene expression) or where multiple modalities have been measured within the same cell. Therefore, an effective diagonal integration method would greatly expand the scope of possible data integration and is enchanting to the community. When we considered the mechanisms that previously published methods use to align modalities, we observed that they are all similarly built upon the foundation of manifold alignment, which projects data from different modalities into a common space while preserving the intrinsic structure within each modality. Therefore, these methods can generally be described in two steps: (1) preserving cell type structure within each modality; and (2) aligning cells across modalities. Each method differs with respect to the representation learning that preserves cell-type structure within each modality and the alignment approach to close the gap between modalities. Thus, they try to solve two problems at the same time and have varying performances of balancing representation learning and modality alignment. Nevertheless, they all share the same underlying principle that aligns data in a low-dimensional manifold.

## Diagonal integration pitfalls revealed with simulated data

**Fig. 1:**
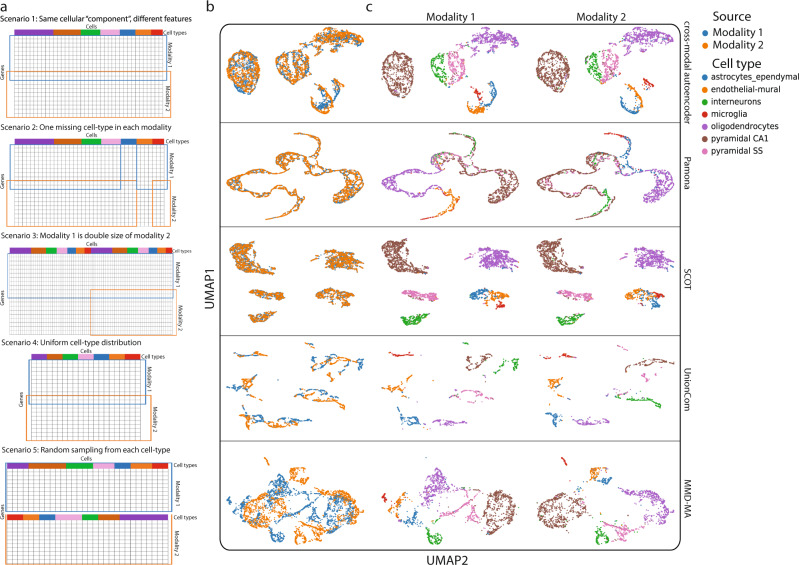
Diagonal integration errors in five scenarios. **a** Five scenarios of simulated multimodal single-cell data, showing how each modality was generated. **b** and **c** Visualization of integration by selected diagonal methods. Cells are colored by modality (**b**) and cell type identity (**c**). The two modalities are split into separate visualizations in **c** to make artificial alignment errors visible. SCIM is not demonstrated due to overall poor representation learning. Alignment outcomes of all five scenarios by each method are shown in Supplementary Figs. 1–6.

Manifold alignment assumes that data from different modalities were generated from a similar distribution or through a similar process. In an ideal experiment, quantification of multi-omics data may satisfy this requirement. But, in reality, there are many unknown variations, and different research labs have different practices of data generation. Therefore, we need to ask how an algorithm distinguishes a true biological alignment that correctly matches the same cell types in different modalities from any other potential artificial alignments. The only judgment the algorithm can make is whether the alignment is the optimal solution. Thus, any artificial alignment that satisfies a mathematical optimum can stand out as the best solution, but will not necessarily represent the accurate biological solution. It seems to lack a mechanism for diagonal algorithms to distinguish a true biological alignment from any artificial alignment without prior knowledge. To demonstrate this pitfall, we illustrate artificial and biologically incorrect alignments resulting from integration applied to a simulated multimodal dataset generated from real single-cell data where the ground truth is known (see “Data availability” and “Code availability” for a full description of this approach). We began with single-cell RNA-seq data from mouse cortex and split the genes into two parts to represent two different “modalities” with different feature spaces, but which come from the same cell population^12^ (Fig. 1). We preserved some shared genes between the two modalities, and both modalities should have a similar power to distinguish the seven cell types. We tested six diagonal methods on five simulated scenarios^6–11^. These methods can distinguish cell types in both modalities separately, and they all align both modalities with no noticeable gap. However, when we investigate cell type correspondence between modalities, we find that these methods all fail at least in one scenario in terms of accurately matching cell types. Since these methods share fundamentally the same mechanism for modality alignment, we conclude that such errors in alignment will be a widespread problem across diagonal methods. We propose that the use of such simulated data should provide a benchmark for future method developments. Developers can investigate in which scenarios their methods may fail and potential reasons for this failure.

## A path forward: partial prior knowledge and benchmarking

Given the outcomes above, we argue that a safe practice in applying diagonal methods is to incorporate certain prior knowledge. Indeed, Yang et al. briefly mentioned that more than one alignment can look equally optimal and incorporating prior knowledge can help deal with issue of artificial alignments^9^. At the same time, Pamona only succeeds in complicated integration when it uses shared cells across modalities^10^. Indeed, when we incorporated shared cells as prior knowledge and ran Pamona again in the five scenarios above, Pamona correctly aligned the same cell types in both modalities, except in scenario 2 (Supplementary Fig. 7). Both publications briefly acknowledge the possibility of artificial alignment we comment on, but this issue has not been highlighted consistently as a key message for those who intend to apply these tools for data integration. Instead, the problem of diagonal integration may come across as solved, and users run the risk of pursuing hypotheses based on erroneous artificial alignment. For example, users could falsely think an enriched signature in one type of data is correlated with an enriched signature in another data type, even though the two aligned cell types in two modalities are not the same.

Considering the incorporation of prior knowledge into future method development, we suggest the following directions here. The first direction is to use partially shared features. Incorporating shared features is feasible for datasets like RNA-seq, ATAC-seq, and other data that are quantified along the linear genome. A pioneering study proposed using partially shared features and extensively benchmarked this hybrid approach with well-established and reliable integration methods^13^. Moving forward, we recommend additional work should continue to investigate how to achieve meaningful integration with minimal shared features and how to identify the minimal set of features that are informative to reveal cell type identities across different modalities. Along with our recommended simulated data above, there is a need for benchmarking datasets that can be used to evaluate the degree and type of shared features that are required to achieve accurate integration. Meanwhile, engineering different modalities to have shared features may not be applicable in cases like integrating gene expression data with chromatin structure data. In such cases, alternative approaches can be constructing a feature-relation matrix, which links features in one modality to possible corresponding features in the other. For example, given an enhancer–promoter contact in Hi-C data, we can hypothesize which gene would be under impact and which histone mark may explain the regulation^14^. However, this approach must be developed with substantial underlying knowledge to support the presumed feature connections. There are also cases in which the construction of feature-relations is not straightforward or lacks experimental support, as in the integration of single-cell omics and single-cell imaging data. This leads to our second recommended direction, using cell anchors or cell labels. In this case, the integration task will be reframed into semi-supervised learning. In recent years, joint-profiling technologies generated multi-omics data at single cell resolution^1–3^, and these joint-profiled single-cell data could serve as reference for learning the integrated space^15^. We envision that combining joint-profiling technologies and diagonal methods would become a standard framework for multimodal single-cell data integration. Further work is also needed to determine how many cells must be profiled by joint methods to represent sufficient complexity to facilitate integration of disparate datasets. Even so, algorithms could misalign cell types that do not show up in the training set, as we showed in cases when we incorporated prior knowledge to run Pamona. Thus, methods should be evaluated for whether they force all data to be aligned to the previously represented cell types or would allow them to be separate.

As diagonal integration gains more attention, the problem of artificial alignment and the two future directions we propose remain major challenges to overcome. When applying diagonal methods in complex situations, the community needs to cautiously evaluate conclusions generated by these methods. In a fast-moving and competitive field, there is strong temptation to show only the advantages of a new method and where it succeeds, making broad claims of general utility while minimizing any potential shortcomings. But it is equally valuable to clearly show scenarios where methods fail, both to inform potential users and to facilitate future research. We encourage the community to contribute additional guidelines for reliable use of diagonal integration methods and to propose additional challenging benchmark tests that will clearly reveal what problems are yet to be solved.

## Supplementary information

Supplementary Information

## Data Availability

The processed single-cell mouse cortex RNA-seq data to generate the simulated two-modality data based on the scRNA-seq data are available at https://github.com/rpmccordlab/cross-domain-simulation.

## Code availability

The code to generate the simulated two-modality data based on the scRNA-seq data are available at https://github.com/rpmccordlab/cross-domain-simulation. To reproduce the analysis results, please follow the analysis code for each integration method in the same GitHub repository.
