# Supplementary material for: Diagonal integration of multimodal single-cell data: potential pitfalls and paths forward
Source: Nat Commun. 2022 Jun 18;13:3505. doi: 10.1038/s41467-022-31104-x (PMC9206644; doi:10.1038/s41467-022-31104-x)
Supplement: Supplementary file 1 — Supplementary Information [file 41467_2022_31104_MOESM1_ESM.pdf]

Supplementary Table 1

Tool	Reference	Principle of alignment
MATCHER	Welch et al., 2017	Finding covariation, and assuming it is unidimensional
MMD-MA	Liu et al., 2019	Minimization of maximum mean discrepancy
UnionCom	Cao et al., 2020	Matching the geometrical distance matrices via GUMA algorithm
SCIM	Stark et al., 2020	Generative adversarial networks
Cross-modal autoencoders	Yang et al., 2021	Generative adversarial networks
Pamona	Cao et al., 2021	Gromov-Wasserstein optimal transport
SCOT	Demetci et al., 2022	Gromov-Wasserstein optimal transport

Diagonal integration tool names and their corresponding references. The principles used to align different modalities are listed in the 3rd column.

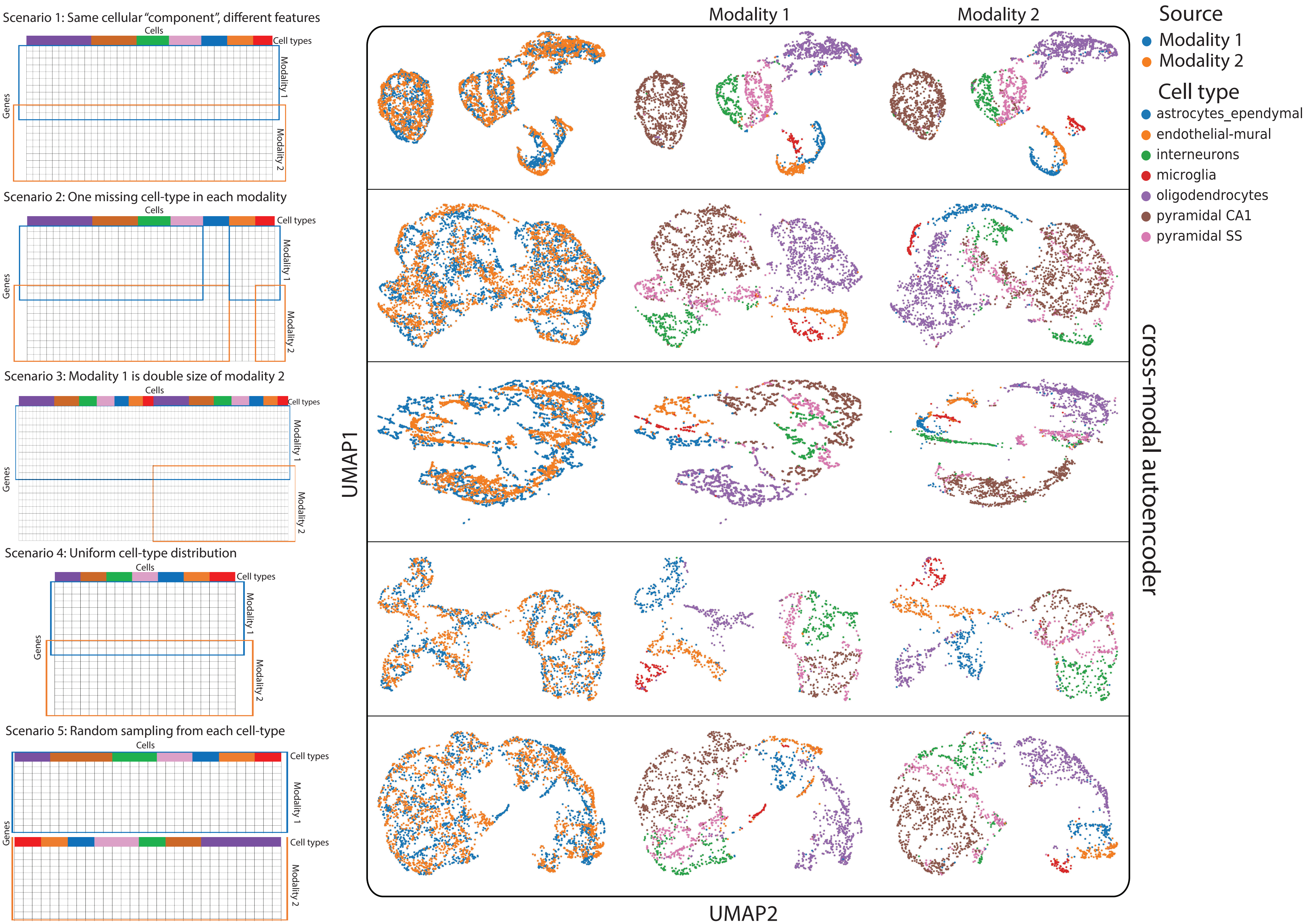

Scenario 1: Same cellular "component", different features
Cells
Cell types
Genes
Modality 1
Modality 2
Scenario 2: One missing cell-type in each modality
Scenario 3: Modality 1 is double size of modality 2
Scenario 4: Uniform cell-type distribution
Scenario 5: Random sampling from each cell-type
Modality 1
Modality 2
Source
Modality 1
Modality 2
Cell type
astrocytes_ependymal
endothelial-mural
interneurons
microglia
oligodendrocytes
pyramidal CA1
pyramidal SS
cross-modal autoencoder
UMAP1
UMAP2

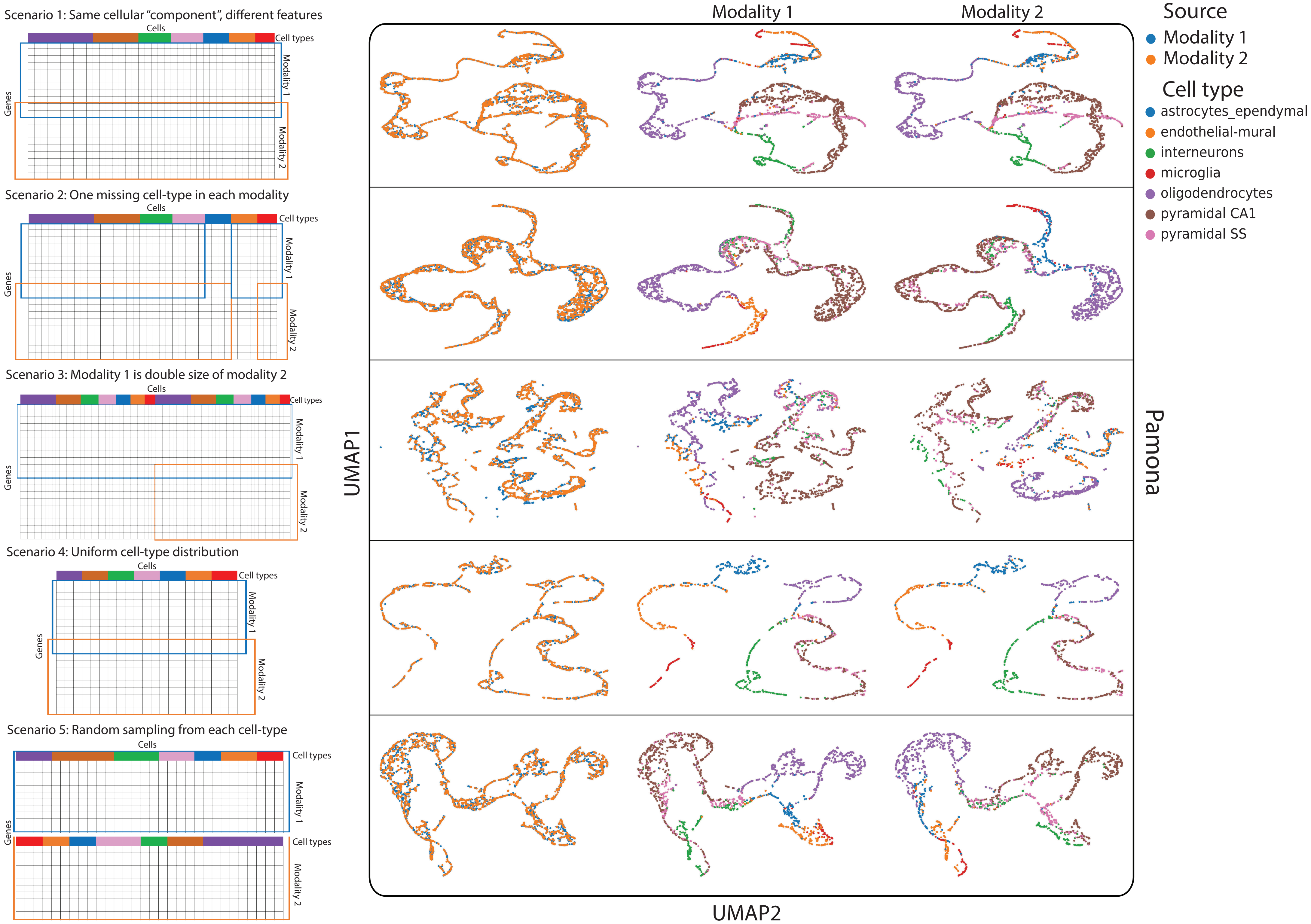

Scenario 1: Same cellular "component", different features
Cells
Cell types
Genes
Modality 1
Modality 2
Scenario 2: One missing cell-type in each modality
Cells
Cell types
Genes
Modality 1
Modality 2
Scenario 3: Modality 1 is double size of modality 2
Cells
Cell types
Genes
Modality 1
Modality 2
Scenario 4: Uniform cell-type distribution
Cells
Cell types
Genes
Modality 1
Modality 2
Scenario 5: Random sampling from each cell-type
Cells
Cell types
Genes
Modality 1
Cell types
Modality 2
Modality 1
Modality 2
UMAP1
UMAP2
Pamona
Source
Modality 1
Modality 2
Cell type
astrocytes_ependymal
endothelial-mural
interneurons
microglia
oligodendrocytes
pyramidal CA1
pyramidal SS

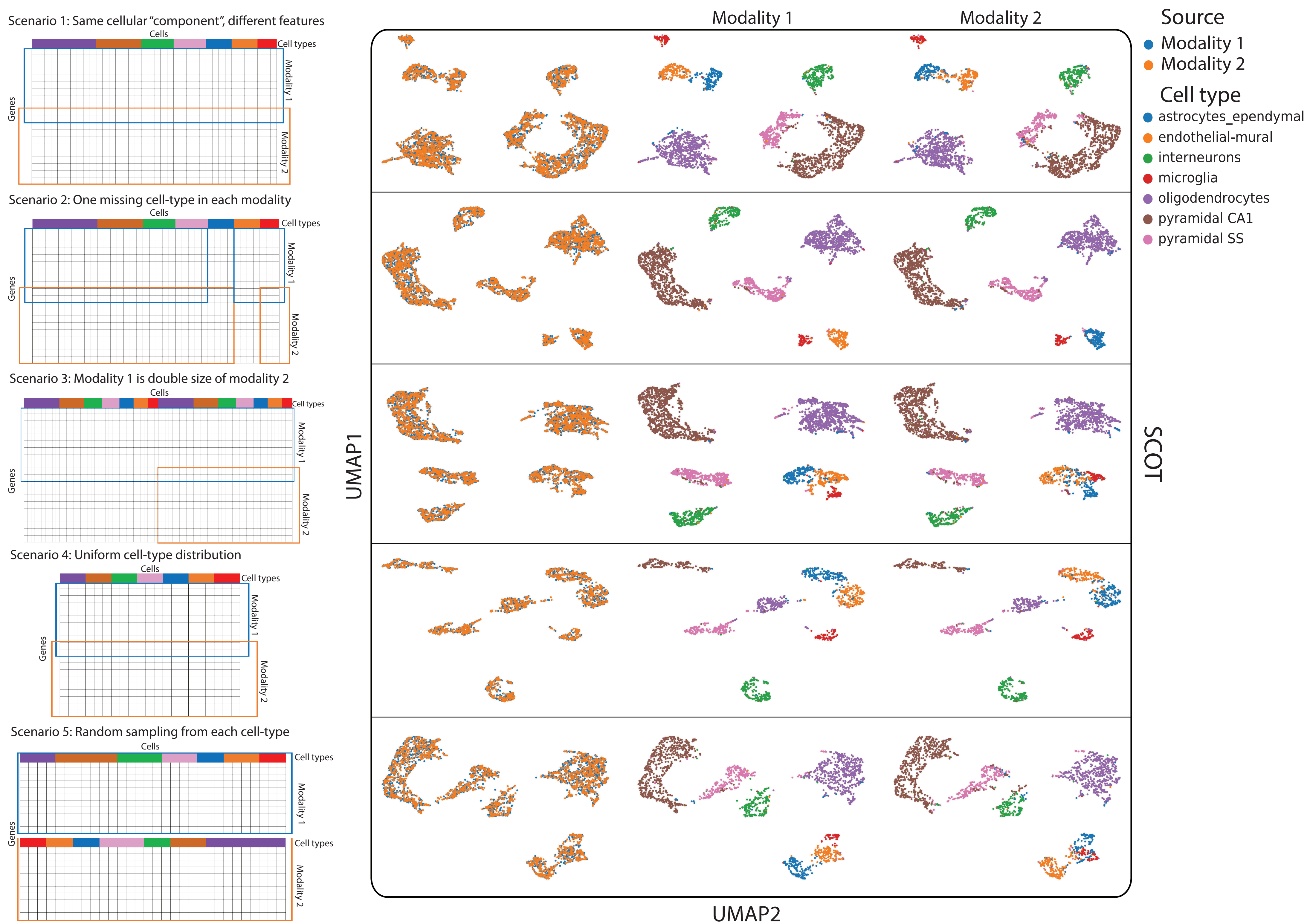

Scenario 1: Same cellular "component", different features
Cells
Cell types
Genes
Modality 1
Modality 2
Scenario 2: One missing cell-type in each modality
Cells
Cell types
Genes
Modality 1
Modality 2
Scenario 3: Modality 1 is double size of modality 2
Cells
Cell types
Genes
Modality 1
Modality 2
Scenario 4: Uniform cell-type distribution
Cells
Cell types
Genes
Modality 1
Modality 2
Scenario 5: Random sampling from each cell-type
Cells
Cell types
Genes
Modality 1
Cell types
Modality 2
Modality 1
Modality 2
UMAP1
UMAP2
SCOT
Source
Modality 1
Modality 2
Cell type
astrocytes_ependymal
endothelial-mural
interneurons
microglia
oligodendrocytes
pyramidal CA1
pyramidal SS

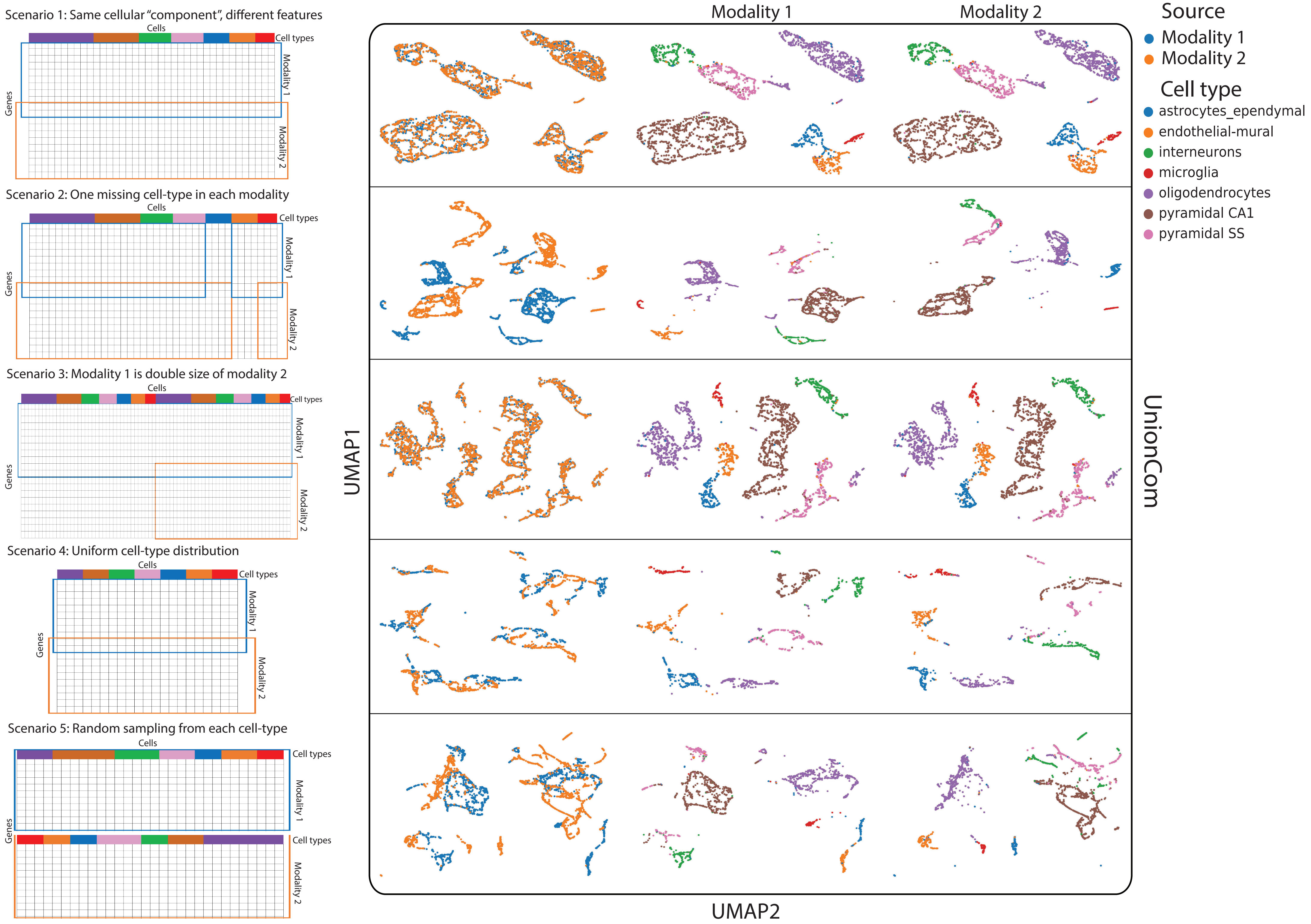
Scenario 1: Same cellular "component", different features
Cells
Cell types
Genes
Modality 1
Modality 2
Scenario 2: One missing cell-type in each modality
Scenario 3: Modality 1 is double size of modality 2
Scenario 4: Uniform cell-type distribution
Scenario 5: Random sampling from each cell-type
Modality 1
Modality 2
UMAP1
UMAP2
UnionCom
Source
Modality 1
Modality 2
Cell type
astrocytes_ependymal
endothelial-mural
interneurons
microglia
oligodendrocytes
pyramidal CA1
pyramidal SS

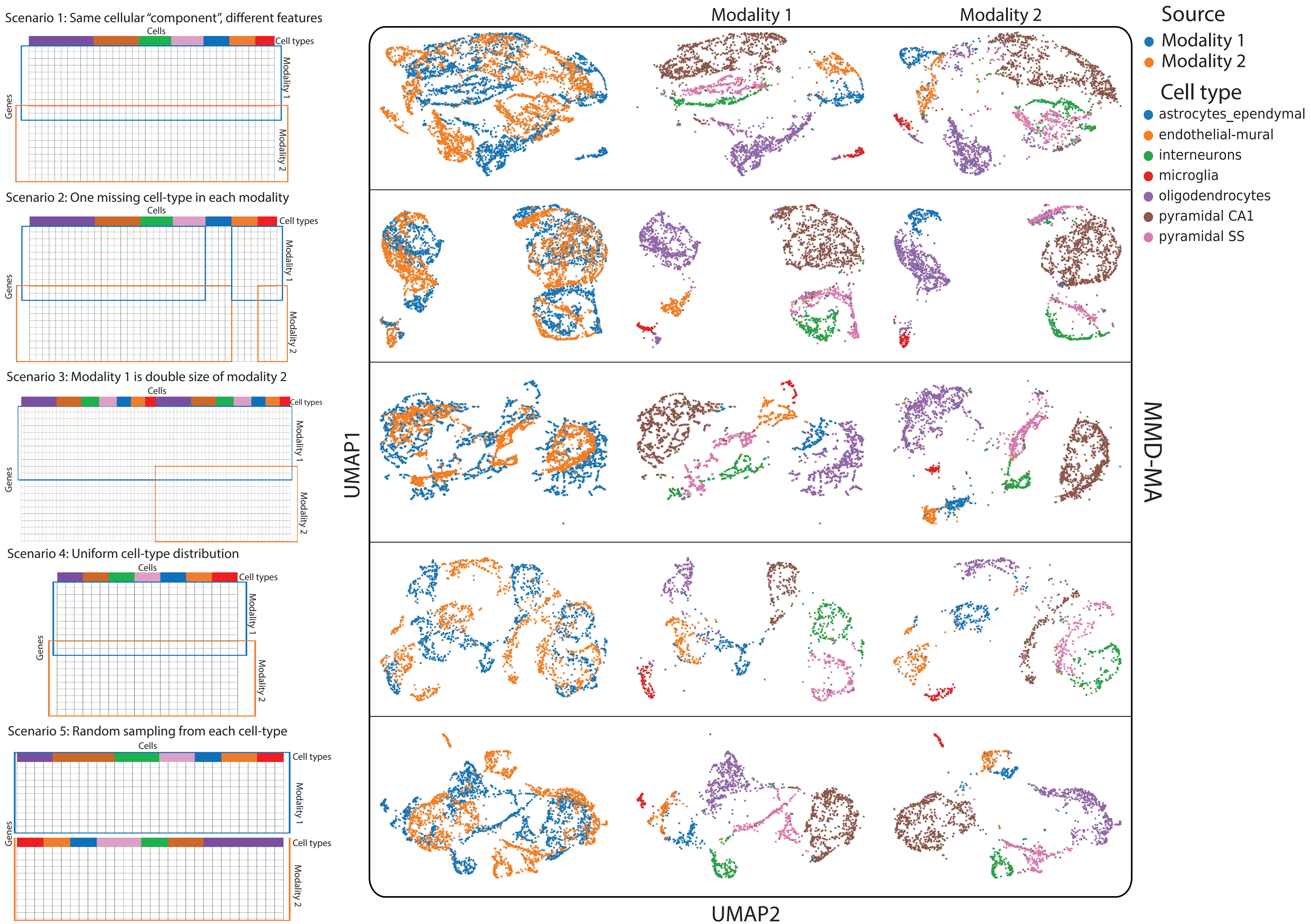

Scenario 1: Same cellular "component", different features
Cells
Cell types
Genes
Modality 1
Modality 2
Scenario 2: One missing cell-type in each modality
Cells
Cell types
Genes
Modality 1
Modality 2
Scenario 3: Modality 1 is double size of modality 2
Cells
Cell types
Genes
Modality 1
Modality 2
Scenario 4: Uniform cell-type distribution
Cells
Cell types
Genes
Modality 1
Modality 2
Scenario 5: Random sampling from each cell-type
Cells
Cell types
Genes
Modality 1
Cell types
Modality 2
Modality 1
Modality 2
UMAP1
UMAP2
MMD-MA
Source
Modality 1
Modality 2
Cell type
astrocytes_ependymal
endothelial-mural
interneurons
microglia
oligodendrocytes
pyramidal CA1
pyramidal SS

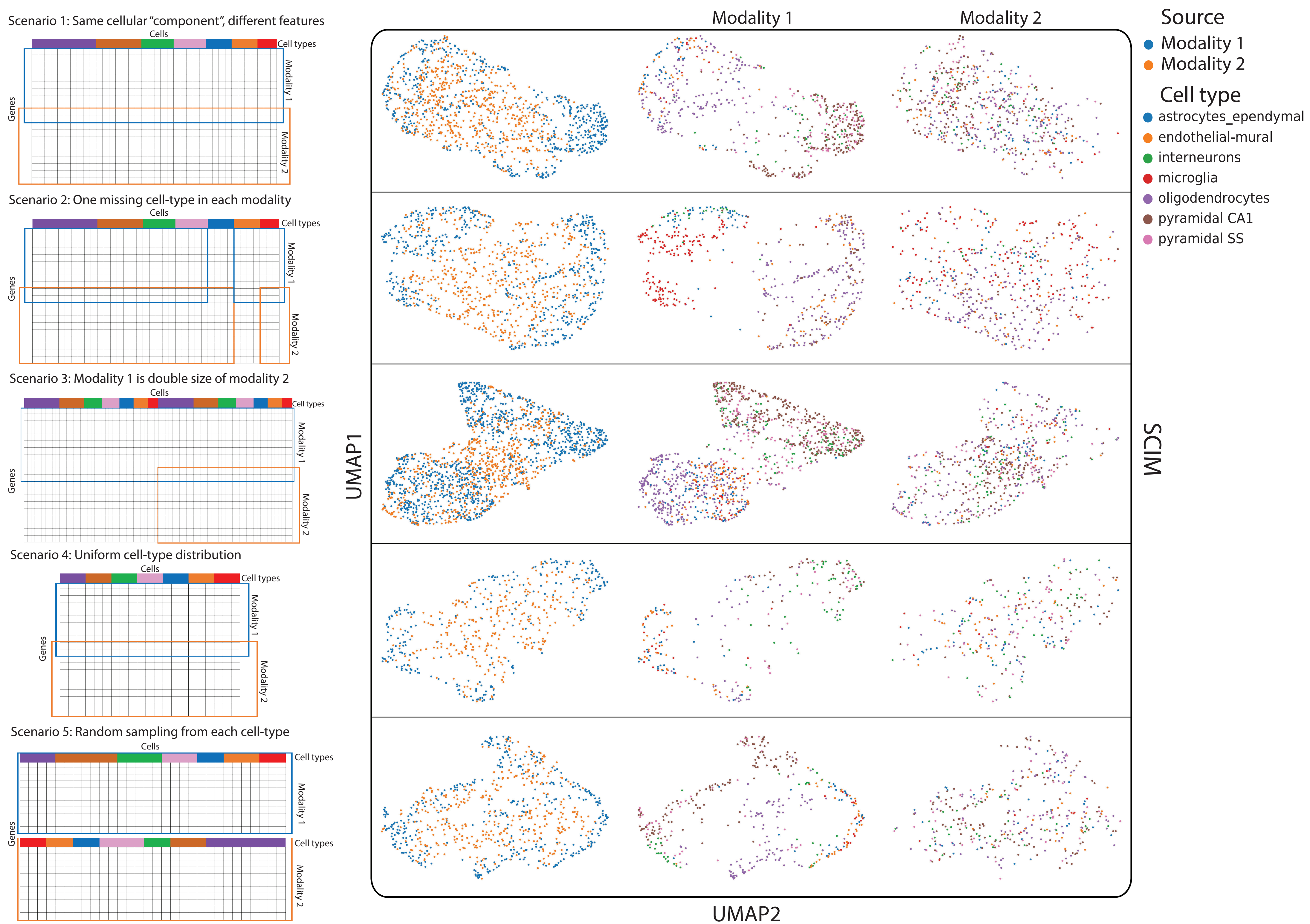

Scenario 1: Same cellular "component", different features
Cells
Cell types
Genes
Modality 1
Modality 2
Scenario 2: One missing cell-type in each modality
Cells
Cell types
Genes
Modality 1
Modality 2
Scenario 3: Modality 1 is double size of modality 2
Cells
Cell types
Genes
Modality 1
Modality 2
Scenario 4: Uniform cell-type distribution
Cells
Cell types
Genes
Modality 1
Modality 2
Scenario 5: Random sampling from each cell-type
Cells
Cell types
Genes
Modality 1
Cell types
Modality 2
Modality 1
Modality 2
UMAP1
UMAP2
SCIM
Source
Modality 1
Modality 2
Cell type
astrocytes_ependymal
endothelial-mural
interneurons
microglia
oligodendrocytes
pyramidal CA1
pyramidal SS

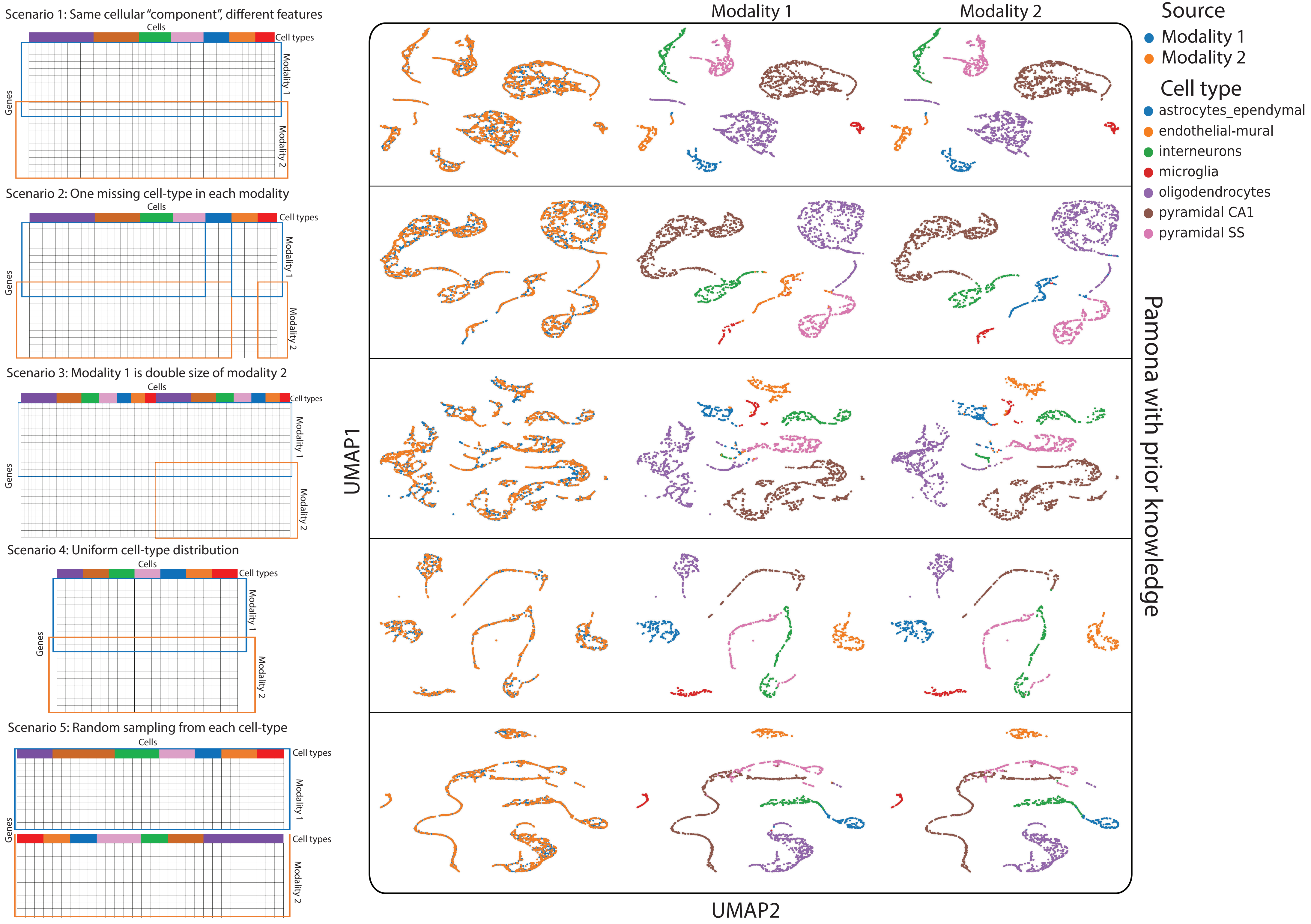

Scenario 1: Same cellular "component", different features
Cells
Cell types
Genes
Modality 1
Modality 2
Scenario 2: One missing cell-type in each modality
Scenario 3: Modality 1 is double size of modality 2
Scenario 4: Uniform cell-type distribution
Scenario 5: Random sampling from each cell-type
Modality 1
Modality 2
UMAP1
UMAP2
Pamona with prior knowledge
Source
Modality 1
Modality 2
Cell type
astrocytes_ependymal
endothelial-mural
interneurons
microglia
oligodendrocytes
pyramidal CA1
pyramidal SS

Supplementary Figure 1: Modality alignment by cross-modal autoencoder across 5 scenarios.

Supplementary Figure 2: Modality alignment by Pamona across 5 scenarios.

Supplementary Figure 3: Modality alignment by SCOT across 5 scenarios.

Supplementary Figure 4: Modality alignment by UnionCom across 5 scenarios.

Supplementary Figure 5: Modality alignment by MMD-MA autoencoder across 5 scenarios.

Supplementary Figure 6: Modality alignment by SCIM across 5 scenarios.

Supplementary Figure 7: Modality alignment by Pamona with prior knowledge across 5 scenarios.
